# Surgery

**Published:** 1903-06

**Authors:** C. A. Morton


### SURGERY.

A child may develop severe abdominal symptoms with fever, and after these have been present some weeks a large collection of pus may be found in the peritoneum. Such cases are obviously quite unlike septic peritonitis due to infection from the appendix; and although, when the abdomen is opened, no evidence of tubercle may be discovered, yet such cases, in the absence of any other explanation, have generally been regarded as such. We now know that such a condition of purulent peritonitis may be due to infection with the pneumococcus; and cases of **pneumococcal peritonitis** have, during the last few years, been described by several writers.

Dr. Fowler, of Edinburgh, gives [2] a very good description of the disease based on the observations of Michaut on eight cases of his own and the collected records of twenty-six other cases, and also on four cases of Stooss, of Bern.[3] He points out that the disease was first described by Netter in 1890; Michaut's work [4] was published in 1901. The disease may take the form of either an encapsuled collection of pus in the peritoneal cavity or a general suppurative peritonitis; but the localised collection of pus is the more common, the other form occurring in only one-third of the cases. The onset is severe; the temperature rises, and abdominal pain and vomiting are present. These symptoms are, of course, present in peritonitis due to other causes; but a symptom which is very suggestive of the pneumococcal form, and to the presence of which attention has

[2] *Scottish M. and S. J.*, 1903, xii. 176.

[3] *Jahrb. f. Kinderh.*, October 20th, 1902. [4] *Thèse de Paris.*

been called by Dieulafoy, is the presence of diarrhœa either at the onset or later on in the course of the disease. Great stress has been laid on its value in distinguishing this from other forms of peritonitis. It is, however, well to remember that in an attack of appendicitis diarrhœa is not rarely present at the commencement, and there may be general peritonitis when we see the case. Quite unlike the course of ordinary septic purulent peritonitis, pneumococcal peritonitis may go on for two weeks or more, and then evidence may be present of a collection of fluid in an encysted form within the abdomen, and if not operated on may burst at the umbilicus. This has happened in several cases. Fowler states that vomiting is a constant symptom. In one case of my own, and in some recorded cases, vomiting was only present at the very beginning of the illness, and did not persist. In a few cases in which the peritonitis is general, the disease runs a rapidly fatal course, and the exudation of pus is never very great, but in others, though general, a large collection of pus forms.

Fowler calls attention to the resemblance between the symptoms of pneumococcal peritonitis, and appendicitis, tuberculous peritonitis and typhoid fever. With regard to the differential diagnosis from appendicitis, he says that although diarrhœa does occasionally occur in appendicitis, its presence should lead one to think of the possibility of the case being one of pneumococcal peritonitis, especially if the patient happen to be a girl (for the disease more frequently occurs in girls); but, as I stated before, diarrhœa is by no means a rare occurrence at the commencement of an attack of appendicitis. The presence of marked rigidity of the abdominal wall over the appendix region, of great tenderness there, or of any swelling localised to this region, or present in the pelvis, and felt either through the abdominal wall or *per rectum*, would, of course, be the important indication of the origin of the disease in the appendix; and in any doubtful case, as Fowler points out, the chances of the disease being appendicitis would be great, for the condition is so common in comparison with pneumococcal peritonitis. Hectic fever and collection of fluid in the abdomen would certainly suggest the presence of tuberculous peritonitis; but Fowler suggests that the sudden onset and rapid course would differentiate between the two conditions. The absence of any nodules, lumps, or bands in pneumococcal peritonitis would also be important, though they may not be discoverable in the tubercular affection. Attention is also called to the similarity between the symptoms of pneumococcal peritonitis towards the end of the first week and typhoid fever. The distended abdomen, the diarrhœa, the slightly enlarged spleen are suggestive of the latter disease; and the sudden onset Fowler considers not a rare occurrence in the typhoid of children. The Widal test would, of course, be valuable.

It is a very disputed question as to the mode of infection of the peritoneum by the pneumococcus. Fowler himself is in favour of the theory of penetration from the intestines, and the presence of diarrhœa as an initial symptom in many of the cases, is certainly suggestive of this source.

The author considers that the prognosis is good if there is an encapsuled collection of pus, and he says Michaut has collected twenty-two cases of this variety, of which twenty-one were operated on with only two deaths; but cases collected from literature are of very little value for such a purpose, as the fatal cases are often not published. But the fatal tendency in the generalised form is certainly indicated by the fact that only two out of eleven cases of Michaut's recovered. Evacuation of the pus in the encysted form, and flushing with saline solution in the generalised variety, should be the treatment.

Nuthall and Billington record[1] five cases of pneumococcal peritonitis: only one case recovered. This was a girl aged 12, from whose peritoneal cavity two pints of pus were evacuated after three weeks' illness with abdominal symptoms. The pus was in the lower part of the peritoneal cavity, and the matted intestines occupied the upper portion. They noted the presence of diarrhœa in three of these cases.

Bryant also has recorded[2] three cases of the generalised form; in two operation was undertaken: all the cases were fatal. The difficulty in the diagnosis of this form of peritonitis in some cases is well illustrated by one of the cases recorded by Bryant, for neither vomiting nor constipation were present, and the condition seemed more like a diaphragmatic pleurisy than a purulent peritonitis. In many of the recorded cases, and in one under my own care, vomiting has been present only at the commencement, or not present at all; and, as already stated, diarrhœa is frequently present.

If the collection of pus within the abdomen is not evacuated it tends to discharge at the umbilicus, and several cases have recovered after this. Dr. Ashby[3] records such a case. The child had double pneumonia, followed by empyema and a large collection of pus in the abdomen, which began to discharge at the umbilicus. In this case pneumococci were not found in the pus from the peritoneum, but they were in the pus from the empyema, and Ashby suggests both conditions were probably pneumococcal in origin. The umbilical opening was enlarged and a drainage-tube inserted, and the child recovered. Dr. Ashby refers to three cases of Broca's,[4] a French physician, in which also spontaneous discharge took place in the umbilicus, with recovery in two of the cases.

The results of **the surgical treatment of ascites due to cirrhosis of the liver** have been investigated by Greenough,[5] who collected

[1] *Birmingh. M. Rev.* 1903, n.s. i. 5. [2] *Brit. M. J.*, 1901, ii. 767.
[3] *Lancet*, 1902, i. 1096. [4] *Arch. de Méd. d. Enf.*, May, 1901.
[5] *Am. J. M. Sc.*, 1902, cxxiv. 979.

the records of 105 cases operated on by the method adopted by Morison,[1] or some modification of it. The operation is undertaken to cure the ascites of cirrhotic liver, and consists in attaching the omentum to the abdominal wall so as to establish a collateral circulation. The author finds that 58 per cent. of the cases were not improved, and of these 29.5 per cent. died within thirty days of the operation. The chief causes of death were shock and exhaustion and peritonitis, but in two cases death was due to intestinal obstruction. Of the forty-two cases improved by the operation, in many the case was published very soon after the operation, and in only nine was continual improvement reported as long as two years after operation. Greenough noted that cases of suture of the omentum between the layers of the abdominal wall did better than those in which there was intra-peritoneal attachment of the omentum.

In considering the advisability of performing this operation, we must not only take into consideration the high mortality and uncertain result in cases which survive, which Greenough has shown to exist, but we must remember that there are channels by which collateral circulation may be established without the production of adhesions. These (described by Sappey) are referred to by Drummond and Morison in their paper [2] in which they advocate the performance of the operation.

Greenough also refers to another method of treating cirrhotic liver—the operation of establishing an opening from the gall-bladder for drainage. Of this operation there are seventeen recorded cases, but there seems no evidence in support of the theory on which it is based, and its beneficial effects are very doubtful.

Although **Volkmann's contraction,** or **ischæmic paralysis,** was described by Volkmann as long ago as 1875, very little is generally known about it. Mr. Leonard Dudgeon [3] has collected the recorded cases, and given us a summary of the pathological changes and symptoms. The essential condition is contraction of the muscles of the forearm leading to flexion of the fingers, and inability to extend them. In the great majority of cases it has come on after the very tight application of splints for an injury, generally a fracture, and in many cases the evidence of the pressure exercised by the splints remains in the form of a pressure-sore near the bend of the elbow. In one case it followed the use of Esmarch's bandage, applied for a little more than an hour, and in another was due to thrombosis of the axillary artery. All the cases are described in Mr. Dudgeon's paper. The term ischæmic paralysis expresses the most frequently accepted views as to its causation. A fibroid change has been found in the muscles in some cases in which an operation has been undertaken to cure the

[1] *Brit. M. J.*, 1896, ii. 728. [2] *Loc. cit.* [3] *Lancet*, 1902, i. 78.

contraction. The contraction may come on within twenty-four hours. Unlike contraction following some forms of paralysis, in this condition it is present from the first. The fingers are strongly flexed at their last two joints, and cannot be extended. The wrist-joint is extended in the less severe cases, flexed in the worst cases. In those cases in which the wrist is not retained in the flexed position, by flexing the wrist the flexed fingers can be somewhat extended. The hand may be fixed in pronation, and the forearm in slight flexion. Some lesion of one or more nerves may or may not be present. If present, sensation may be affected. A nerve lesion must be regarded as a complication, and not as the cause of the disease, which is of the nature of a "myositis" or sclerosis of the flexor muscles. The author refers to a statement of Volkmann, that the prognosis in this condition is bad; but he has had experience of several cases which in the course of a few years quite recovered. In the treatment of this condition, forced extension of the fingers does not seem to lead to any good result. In one case portions of the bones of the forearm were excised in order to lessen the tension of the flexor muscles: this result was obtained, but the bone only united by fibrous tissue. The best operative procedure seems to be lengthening the contracted tendons by splitting them longitudinally and then uniting one section to the end of the other. Some of the cases which have recovered in a year or two have only been treated by massage.

Mr. Montgomery has published a valuable paper [1] on his experience of twenty-five cases of **tendon grafting for paralytic talipes.** He prefers not to divide the tendon of the active muscle, except in the case of the extensor of the great toe, the action of which can, he thinks, be performed by the tendon of the extensor brevis, but advises that a slip should be taken from the tendon of the active muscle and attached to the divided end of the tendon of the paralysed muscle, for he thinks this a better plan than grafting it on to the side of the tendon of the paralysed muscle, or the grafting of the cut end of the tendon of the paralysed muscle on to the side of the tendon of the active muscle. His preference for this method is due to his experience that, if adopted, quicker and better results are obtained. As he points out, however, one must be convinced that no spontaneous improvement can take place before dividing the tendon of the paralysed muscle. In cases in which he has grafted a slip from the gastrocnemius or soleus to the paralysed dorsi-flexors improvement was very slow. He was here grafting the tendon of an antagonistic group of muscle, and in such a case a really good result is only with difficulty obtained, but he says that, although slowly, such cases did steadily improve after operation.

C. A. MORTON.

[1] *Med. Chron.*, 1902-3, 4 s., iv. 97.

